# Lipome intramédullaire: à propos d'une observation

**DOI:** 10.11604/pamj.2018.31.244.9453

**Published:** 2018-12-26

**Authors:** Soukaina Wakrim, Najwa Touil, Ousmane Traore, Omar Kacimi, Nabil Chikhaoui

**Affiliations:** 1 Service de Radiologie des Urgences CHU Ibn Rochd Casablanca, Maroc

**Keywords:** Lipome intramédullaire, imagerie par résonance magnétique nucléaire, intramédullaire non dysraphique, Intramedullary lipoma, nuclear magnetic resonance imaging, non dysraphic intramedullary

## Abstract

Les lipomes intramédullaires sont des lésions bénignes rares qui représentent environ 1% de l'ensemble des tumeurs de la moelle épinière. Nous rapportons un nouveau cas de lipome intramédullaire non dysraphique confirmé histologiquement. Il s'agissait d'une patiente âgée de 46 ans ayant bénéficié d'une biopsie chirurgicale pour un lipome médullaire il y a 6 mois. Nous ne disposons pas de documents radiologiques antérieurs à cette chirurgie. Elle présente actuellement des rachialgies, des troubles sensitifs, des troubles de la marche et une faiblesse musculaire d'aggravation récente. L'IRM médullaire objective une formation en hypersignal T1 et T2 bien limitée de 8cm x 2,5cm prenant le cône terminal. L'imagerie par résonance magnétique occupe une place primordiale dans l'exploration des lipomes intramédullaires, elle permet le diagnostic précoce et ainsi une prise en charge chirurgicale avant la survenue de complications neurologiques irréversibles.

## Abstract (en)

Intramedullary lipomas are rare benign lesions accounting for approximately 1% of bone marrow cancers. We report a new case of histologically confirmed non-dysraphic intramedullary lipoma. The study involved a 46-year old female patient who had undergone surgical biopsy for medullary lipoma 6 months before and who did not have radiological reports prior to this surgery. She suffered from rachialgias, sensory disorders, difficulties to walk and recently aggravated muscular weakness. Medullary MRI objectified well-defined mass presenting hypersignal on T1 and on T2 measuring 8cm x 2.5cm in the conus terminalis. Magnetic resonance imaging is the gold standard for the exploration of intramedullary lipomas. It allows early diagnosis as well as surgical management before the onset of irreversible neurological complications.

## Introduction

Le lipome intramédullaire non dysraphique est une lésion bénigne rare qui représente environ 1% de l'ensemble des tumeurs de la moelle épinière. Les lipomes véritablement intramédullaires sont très rares. Ils sont décrits dans la littérature sous forme de rares cas cliniques isolés [1-8]. Avec l'avènement de l'imagerie par résonance magnétique le nombre de cas publié a significativement augmenté depuis 1995. Nous rapportons l'observation d'une patiente atteinte d'un lipome intramédullaire non dysraphique dorsolombaire.

## Patient et observation

**Figure 1:**
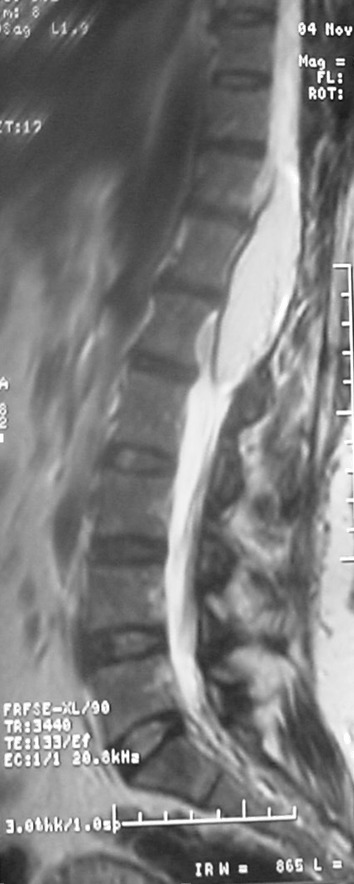
image IRM en pondération T2, montrant un hyper signal du lipome intra médullaire dorsolombaire s'étendant de D11 à L1

**Figure 2:**
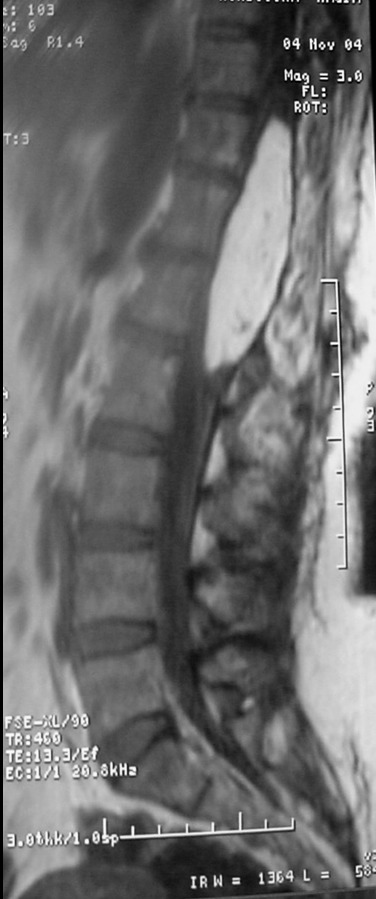
image IRM en pondération T1, montrant un hyper signal du lipome intra médullaire dorsolombaire s'étendant de D11 à L1

Une femme âgée de 46 ans ayant bénéficié d'une biopsie chirurgicale pour un lipome médullaire il y a 6 mois. Nous ne disposons pas de documents radiologiques antérieurs à cette chirurgie, qui accuse depuis 4 ans des troubles sensitifs et douloureux des deux membres inférieurs compliqués il y a une année par un steppage du membre inférieur gauche. À l'examen physique, il existait une faiblesse musculaire des membres inférieurs, associée à une spasticité et une hyperréflexie, un clonus du pied droit, un signe de Babinski, des troubles de la sensibilité profonde des pieds et un niveau lésionnel sensitif en D11 avec hypoesthésie superficielle à la douleur et à la température de la partie sous-jacente du corps. Cette patiente a bénéficié d'une biopsie chirurgicale, l'analyse histologique montrait que la tumeur biopsiée était composée en totalité de tissu adipeux mature. Une IRM a été faite dans le cadre d'une surveillance radiologique de la lésion. Elle montrait l'existence d'un lipome intramédullaire, s'étendant de D11 à L1, responsable d'un hypersignal sur les séquences T1 et T2 (Figure 1, Figure 2), mesurant 8cm de grand axe et 2,5cm de diamètre antéropostérieur. L'injection de gadolinium ne modifiait pas le signal.

## Discussion

Le lipome intramédullaire non dysraphique est une tumeur rare représentant moins de 1% de toutes les tumeurs intramédullaires [1-5], touchant dans la majorité des cas l'adulte jeune, exceptionnellement l'enfant. Sans prédominance de sexe [3]. Le siège de prédilection reste la moelle cervicale et dorsale [2-8]. Après un délai diagnostic assez long (> 8 mois), les patients se présentent le plus souvent avec un tableau clinique de compression médullaire d'évolution lente, et pouvant simuler une compression d'origine maligne [8]. Les principaux signes cliniques sont des rachialgies, des troubles de la marche, la faiblesse musculaire et les troubles sensitifs subjectifs et les troubles sphinctériens [2, 3, 5]. Le niveau de ces signes dépend du siège de la tumeur. On retrouve habituellement des symptômes anciens d'aggravation récente. Dans la plupart des cas rapportés, l'atteinte neurologique est sévère lors de l'évaluation initiale. Les radiographies standard de face et de profil qui font partie du bilan systématique devant tout syndrome rachidien peuvent montrer des anomalies témoignant d'une tumeur intrarachidienne d'évolution lente comme un élargissement du canal médullaire et des images d'encoche vertébrale (scalloping) [7]. L'IRM est l'examen de référence pour le diagnostic [2-4, 9]. Elle montre un signal graisseux en pondération T1 et T2, sans modification après injection de gadolinium. Son apport est considérable, car elle permet un diagnostic plus précoce avant la survenue de complications neurologiques irréversibles et guide la chirurgie pour éviter les difficultés de l'exérèse liées aux rapports très étroits entre le lipome et les racines nerveuses empêchent parfois la résection tumorale complète. Les patients chez lesquels la résection du lipome intramédullaire n'a pu être que partielle doivent être informés qu'une prise de poids ou une grossesse peut aggraver les signes neurologiques [7]. Les corticostéroïdes endogènes ou exogènes interviennent dans la croissance des tumeurs lipomateuses de différents sièges anatomiques, y compris celles de la moelle épinière [9].

## Conclusion

Les lipomes intramédullaires non dysraphiques sont des tumeurs bénignes rares de l'adulte jeune, évoluant lentement vers l'aggravation. De topographie essentiellement cervico-dorsale, leur diagnostic doit se faire précocement avant le stade de myélopathie évoluée. L'IRM reste l'examen de choix, mettant en évidence un processus intramédullaire de nature graisseuse sans signes de dysraphismes spinaux. Le traitement est chirurgical consistant à une exérèse tumorale décompressive tout en évitant l'interface lipome-moelle, source d'aggravation postopératoire. L'évolution est favorable et les récidives sont exceptionnelles.

## Conflits d’intérêts

Les auteurs ne déclarent aucun conflit d'intérêts.
